# Understanding Citrus Viroid Interactions: Experience and Prospects

**DOI:** 10.3390/v16040577

**Published:** 2024-04-09

**Authors:** Yafei Wang, Yan Shi, Honglian Li, Jiaxin Chang

**Affiliations:** College of Plant Protection, Henan Agricultural University, Zhengzhou 450002, China; shiyan00925@126.com (Y.S.); honglianli@sina.com (H.L.); changjiaxin@163.com (J.C.)

**Keywords:** citrus viroids, antagonism, synergism, RNA silencing, vd-siRNA

## Abstract

Citrus is the natural host of at least eight viroid species, providing a natural platform for studying interactions among viroids. The latter manifests as antagonistic or synergistic phenomena. The antagonistic effect among citrus viroids intuitively leads to reduced symptoms caused by citrus viroids, while the synergistic effect leads to an increase in symptom severity. The interaction phenomenon is complex and interesting, and a deep understanding of the underlying mechanisms induced during this viroid interaction is of great significance for the prevention and control of viroid diseases. This paper summarizes the research progress of citrus viroids in recent years, focusing on the interaction phenomenon and analyzing their interaction mechanisms. It points out the core role of the host RNA silencing mechanism and viroid-derived siRNA (vd-siRNA), and provides suggestions for future research directions.

## 1. Introduction

Viroids are currently the simplest known pathogens that infect plants. They are long non-coding RNA molecules composed of 246–434 nucleotides (nts) and have a single-stranded, covalently closed circular genome [1]. Viroids infect plant cells through mechanical means, replicate and move within the host, and can cause serious diseases in vegetables, flowers, and fruit trees [2,3,4]. Citrus is a perennial woody plant in the *Rutaceae* family that is infected by various viroids. So far, citrus is a natural host of at least eight viroid species, including Citrus exocortis viroid (CEVd), Citrus bent leaf viroid (CBLVd), Hop stunt viroid (HSVd), Citrus dwarfing viroid (CDVd), Citrus bark cracking viroid (CBCVd), Citrus viroid V (CVd-V), Citrus viroid VI (CVd-VI), and Citrus viroid VII (CVd-VII). CEVd is the pathogen of citrus exocortis disease and is a member of the genus *Pospiviroid*. CBCVd, formerly known as *Citrus viroid IV*, belongs to the genus *Cocaviroid*. HSVd can induce stem pitting and bark splitting in susceptible citrus varieties and belongs to the genus *Hostuviroid*. In addition, CBLVd, CDVd, CVd-V, CVd-VI, and CVd-VII all belong to the genus *Apscaviroid*. These citrus viroids are widely present and often infect citrus orchards around the world in combination, and exhibit complex interactions during mixed infection.

There are two opposite types of interactions among citrus viroids co-infecting the same plant, leading to antagonistic or synergistic effects [5,6,7,8,9]. As a natural host of the abovementioned viroid, citrus constitutes an excellent platform for exploring the complicated mechanism of inter-viroid interactions. The citrus exocortis disease caused by CEVd is one of the most important viroid diseases threatening the global citrus industry. Previous field symptom observations have confirmed the cross protection of CBCVd against CEVd, as the combination of these two types of viroids can significantly reduce the symptoms of CEVd on sensitive rootstocks [10]. When citrus viroids belonging to the genus *Apscaviroid* infect citrus trees with citrange as rootstocks, they cause plant dwarfing and reduced yield [10,11]. Observations of field symptoms revealed that even without CEVd, the combined infection of other types of citrus viroids can still cause typical symptoms of exocortis disease on citrange rootstocks. According to biological testing results, when other species of viroids co-infect “Etrog” citron, they can also cause symptoms similar to CEVd. The co-infection of CBLVd and CVd-V, or the co-infection of CDVd and CVd-V in “Etrog” citron or field-grown citrus trees, results in synergistic effects among viroids and in more severe symptoms than simple superimposition [9,10]. Therefore, it is necessary for us to conduct a systematic and in-depth study on the antagonistic and synergistic effects observed during viroid co-infection, which is of great theoretical and practical significance for us to further understand the molecular mechanisms of viroid pathogenicity for the prevention and control of viroid diseases.

## 2. Sequence Characteristics of Citrus Viroids

CEVd (371 nts, reference sequence NC001464) has a rod-shaped secondary structure [12]. The rod-shaped structure consists of five structural domains: left terminal region (TL), pathogenic region (P), central region (C), variable region (V), and right terminal region (TR) [13]. Visvader et al. [14,15] used tomato as an experimental host to classify CEVd into severe isolates and mild isolates, with at least 26 nt differences between them. These two types of CEVd isolates induce different symptoms in *Gynura aurantiaca* [16], but there is only a slight difference in symptoms on the rootstock of trifoliate orange [11]. The relationship between CEVd sequence and pathogenicity is more complex than expected. Murcia et al. [17] identified an unusual strain (CEVd-COL) that caused severe symptoms in tomatoes, but unexpectedly caused extremely mild symptoms in the “Etrog” citron. The use of site-specific mutagenesis resulted in two nucleotide substitutions in the down chain of the P domain of CEVd-COL and the resulting CEVd-MCOL variant could cause severe symptoms in the “Etrog” citron, indicating that the pathogenic determinants of CEVd are actually host-dependent. CEVd can generate larger molecules by repeating specific sequences in rod-shaped secondary structures. A CEVd mutant CEVd D92 with 92 nt replicates was identified in hybrid tomatoes (*Solanum lycopersicum* × *S. peruvianum*) [18], and a CEVd mutant CEVd-D96 with 96 nt replicates was identified in eggplant [19]. These enlarged CEVd molecules have been identified as stable CEVd mutants and still possess stable rod-shaped secondary structures. However, these CEVd isolates have not been reported in citrus species.

HSVd (299 nt, reference sequence AY594203) is a typical representative of the genus *Hostuviroid* in the family *Pospiviroidae*. HSVd contains a genus-specific central conservative region and an end-conservative hairpin, but lacks an end-conservative region [20]. CBCVd (284 nt, reference sequence NC003539) belongs to the genus *Cocaviroid* in the family *Pospiviridae*. CBCVd is infectious in both citrus and hops, and its genome has sequence homology with HSVd in the left terminal region, while it has high sequence homology with CEVd in the right terminal region. Therefore, CBCVd is considered a chimeric recombinant of CEVd and HSVd [21,22]. In addition, Wang et al. [23] detected the CBCVd Pakistani isolates named CBCVd-lss with significant sequence differences from the right-handed end of the CBCVd standard sequence in Pakistan citrus samples. The genus *Apscaviroid* includes five citrus viroids, namely, CBLVd (318 nts, reference sequence M74065), CDVd (327 nts, reference sequences S76452 and 324 nt, reference sequence AF184147), CVd-V (294 nts, reference sequence EF617306), CVd-VI (330 nts, reference sequence AB019508), and CVd-VII (368 nts, reference sequence KX013551) [24,25]. Given the widespread distribution of citrus viroids worldwide, a large number of complete genome sequences of citrus viroids can be obtained from sequence databases. In addition, an analysis of the offspring population after the inoculation of plants with a single sequence revealed the presence of mutant sequences in the citrus viroid offspring population, further increasing the sequence diversity of citrus viroids [26,27].

## 3. The Pathogenesis of Citrus Viroids

Although citrus viroids have been discovered due to their strong pathogenic ability, not all citrus viroid infections have obvious symptoms. Citrus viroids typically cause stunted development of infected plants, accompanied by other symptoms including leaf curling, the distortion of leaves and flowers, vein necrosis, bark disorders, fruit changes, sterility, and rapid aging. The symptoms of citrus viroids are usually enhanced by high temperatures, which is exactly the opposite of most plant viruses. Škorić et al. [28] conducted experiments using two stable CEVd variants and observed that as the daytime temperature increased from 35 °C to 40 °C, the symptoms caused by the weak CEVd strain intensified, and this effect was reversible. At the micro level, the cytopathic effects caused by viroid infection include the proliferation of cytoplasmic membranes, the distortion of cell walls, chloroplast abnormalities, and the formation of electron-dense deposits [29]. It is not yet clear how these micro effects are associated with symptoms at the macro level. Many symptoms associated with citrus viroid infection are often associated with hormonal metabolic disorders. Changes in hormone regulatory pathways were found in tomatoes infected with CEVd [30] and cucumbers infected with HSVd [31]. Citrus viroid infection can also affect host macromolecules. According to reports, two pathogenesis-related (PR) proteins have changed in *Gynura aurantiaca* infected with CEVd [32]. Citrus viroids cannot encode proteins, and their replication relies entirely on host factors. In the absence of peptides encoded by viroids, diseases were initially considered as the result of direct interactions between viroids and cellular components [33]. Later, regulatory pathways similar to RNA silencing were discovered, and the indirect interaction between viroids and hosts was gradually understood [4].

In order to defend themselves, plants have evolved strategies to resist viroid infections. RNA silencing is the host plant’s defense mechanism mainly against viral invading pathogens [34,35]. Viroid replication can trigger plant RNA silencing mechanisms. RNA silencing is initiated by double-stranded RNA (dsRNA), which is cleaved into small RNAs (sRNAs) by Dicer-like proteins (DCLs) in plants [36]. One strand of the double-stranded sRNA guides the RNA silencing complex (RISC) to inactivate complementary RNA or DNA. Viroids in plants are usually inhibited by a combination of DCLs and RISCs [34]. Mature viroid RNA molecules are directly targeted by DCLs and degraded into viroid-derived siRNA (vd-siRNA) [37]. In addition, viroid replication intermediates in the form of dsRNA can also be processed by DCLs to obtain vd-siRNA. Experiments have shown that vd-siRNAs play a crucial role in resisting viroid invasion of plants [38,39,40]. Vd-siRNA can reduce the expression of reporter genes in infected plants and viroid titers [37,41]. Vd-siRNA can be loaded onto Argonaute proteins (AGOs) to target the mature viroid genome or the replication intermediates [42]. RNA silencing also mediates viroid pathogenicity. Vd-siRNA not only guides RISC to counteract viroid RNA but also targets homologous host transcripts, inactivating them and ultimately leading to symptoms through signal transduction cascades [43]. Therefore, the symptoms induced by viroids may be a result of host gene silencing [44]. In addition to activating post transcriptional gene silencing (PTGS), viroids also trigger RNA-directed DNA methylation (RdDM) of homologous sequences [45]. In cucumber, HSVd infection causes dynamic changes in DNA methylation of ribosomal RNA genes, indicating that epigenetics may affect viroid symptoms [46]. The molecular mechanisms by which plants defend against attacks from different pathogens (fungi, bacteria, viruses, and viroids) share many common features, such as reduced photosynthesis. Most host genes affected by viroid infection are also affected during other biological stresses, indicating that one or more shared signal transduction pathways and gene regulatory networks may be involved in the host’s regulation of viroid infection. The pathogenic mechanism of viroids is complex. It is an interesting question how viroid infections spread from initial interactions to affect different metabolic pathways. An in-depth analysis of tomatoes infected with CEVd suggests that post transcriptional changes may involve translation inhibition and sRNA-mediated mRNA degradation [47]. A large amount of sRNA sequence analysis reveals changes in host miRNA levels after viroid infection [48]. After infecting plants, vd-siRNAs similar to endogenous miRNAs and siRNAs are produced [49,50]. Vd-siRNAs may have the same function as endogenous miRNAs and siRNAs, which can be assembled into RISCs to inactivate endogenous mRNA. Therefore, it is speculated that vd-siRNAs are a key factor in the pathogenicity of viroids [43].

## 4. Response of Citrus Hosts to Viroid Infection

The non-coding property of viroids implies that symptoms associated with viroid infection are caused by changes in host gene expression. RNA silencing is an important mediator for the interaction between viroids and hosts. Based on deep sequencing technology, the expression levels of gene products containing potential binding sites of vd-sRNA can be analyzed. It is speculated that the proteins that can interact with citrus viroids mainly include DCL1-4 and corresponding AGOs, nuclear DNA ligase 1, and RNA-dependent RNA polymerases (RDRs). Through the study of host protein changes caused by CEVd and PSTVd infections, it was found that the PR protein induced by viroids is a universal component of the host response to pathogen attacks [51]. Almost all studies have reported a decrease in photosynthesis, possibly to save the energy needed for host defense reactions [52]. Respiratory and secondary metabolism increase in many pathological systems. The proteins accumulated under pathogen attack include PR proteins, antioxidant enzymes, and certain protein components related to translation. Lisón et al. [47] observed a direct correlation between changes in defense-related proteins at the protein and mRNA levels in tomatoes infected with CEVd.

Functional genomics based on microarrays has been widely used to analyze overall changes in host transcriptional levels during plant viroid interactions [53]. In order to clarify how the expression of host genes changes over time during viroid infection, Rizza et al. [54] compared the expression of “Etrog” citron genes in the early (pre symptomatic) and late (post symptomatic) stages of CEVd infection, and found that CEVd infection caused significant changes in chloroplast, cell wall, peroxidase, and transporter protein activity. A transcriptome sequencing analysis conducted by Wang et al. [55] on “Etrog” citron samples infected with CEVd showed that CEVd infection can trigger host plant basic defense responses, disrupt plant hormone signal transduction, induce the expression of key genes in RNA silencing pathways, and affect cell wall and secondary metabolite synthesis. CDVd is often described as a tree regulator with dwarfing effects, and most of the transcriptomic changes it induces occur in the scion rather than in the rootstock [56]. Tessitori et al. [57] used CDVd-citron as a model to study the molecular mechanisms of interaction between viroids and host plants. They identified 18 upregulated and downregulated genes mainly related to cell wall structure, amino acid transport, signal transduction, and plant defense response. It is particularly noteworthy that RNA silencing inhibitors involved in the pathogenic response to viroids are also upregulated genes. Vd-siRNA may act as an inducer of symptom expression by mediating host mRNA cleavage or the methylation of promoter regions. Several research teams have reported the changes in host miRNA after viroid infection [58,59,60,61]. Numerous potential target genes for transcription factors and other regulatory proteins may occupy important positions in viroid–host interactions.

## 5. Antagonistic Effect among Citrus Viroids

Antagonistic phenomena are common in fungi, bacteria, viruses, and viroids [62,63]. The antagonistic effect is also known as cross protection in virology [64,65,66,67]. Cross protection measures have been applied to greenhouses and fields [68,69,70], and the tolerance of a plant against virulent virus strains is induced by systemic infection with a mild strain [71,72,73]. The antagonistic effect among viroids refers to the ability of viroids to infect the host being influenced by the pre-infection of other strains of the same or closely related viroids [66,74]. Specifically, when plants pre-infected with a weak viroid strain are inoculated with a strong strain of the same viroid, the typical symptoms of the strong strain and its RNA accumulation level will be blocked or weakened for a period of time.

There are antagonistic effects among closely related viroids and different strains of the same viroid [75]. CEVd can cause serious symptoms such as bark cracking, shedding, and even root rot on citrus rootstocks, such as citrange and other varieties. Duran–Vila and Semancik [64] demonstrated that mild strains of CEVd can provide significant protection against challenges with severe strains. The level of protection depends on the inoculation interval between mild and severe strains. Vernière et al. [10] found that trees infected with a mixture of CEVd and CBCVd showed an antagonistic effect in terms of bark scaling and cracking symptoms. CEVd can cause severe tree dwarfing and bark cracking of trifoliate orange rootstocks, both of which are characteristics of exocortis disease. When the viroid mixture contains CBCVd, the symptoms of bark cracking are alleviated or even suppressed. The impact of CBCVd on citrus growth and fruit yield is minimal [11]. Therefore, CBCVd can serve as an antagonistic factor for the symptoms of CEVd, and CBCVd can reduce or inhibit the effects of CEVd on citrus growth and yield. It should be noted that the main and secondary structures on the right side of the CEVd and CBCVd genomes are almost identical, indicating that the relationship between the two may be closer than indicated by taxonomy [22,76].

The RNA silencing mechanism in plants can be induced by dsRNA and involves three basic components: DCLs, RDRs, and AGOs [77,78,79]. Previous studies have shown that viroid RNA is an activator and target of RNA silencing in hosts [53,80,81,82]. On the contrary, the subsequently generated vd-siRNA also plays a crucial role in the pathogenesis of the viroid by targeting the RNA transcripts of host genes [83,84]. Gene silencing largely explains why cross protection only occurs among viroids with sequence homology. Assuming that the DCLs of plants act on the mild viroid that was first inoculated to produce corresponding vd-siRNAs, they activate the RISC, thereby providing corresponding protection against the severe viroid infection that follows. We have reasons to believe that the cross protection of viroids depends on the sequence homology of vd-siRNAs entering RISC. Due to the common sequence and structural characteristics between the CEVd and CBCVd genomes [76], the antagonistic effect of CBCVd on CEVd may be related to RNA silencing and vd-siRNA.

The titer of viroids can be co-regulated by DCLs and the RISC. DCLs process genomic RNA and the replication intermediates of viroids, and the resulting siRNAs are assembled into RISC to target and degrade the viroid RNA [2]. A similar mechanism can explain the antagonism among citrus viroids. The siRNAs produced during the mild viroid inoculation target the severe viroid RNA that was later inoculated. Symptoms of citrus viroids often appear after 3–8 years of field colonization on susceptible rootstocks. Qiu et al. [85] shortened the research period to 1–3 months by inoculating “Etrog” citron plants with viroid transcripts in vitro. A study by Qiu et al. [85] pointed out that early inoculation of CBCVd can weaken the symptoms of leaf roll and necrosis caused by CEVd, and reduce the accumulation of CEVd. Sequence analysis revealed that CBCVd and CEVd have highly similar sequences in the right terminal region, and both produce a large amount of vd-siRNA in the right terminal region. The vd-siRNA lengths from CEVd and CBCVd are mainly 21 nt and 22 nt, and they have similar 5′ base preferences toward U and C corresponding to AGO1 or AGO5, respectively [85]. Further research has found that siRNA expressed in the right terminal region of CBCVd can directly degrade the transcripts of CEVd in vivo through the host’s RNA silencing mechanism, leading to a decrease in the accumulation of CEVd [85].

The antagonistic effect of CBCVd and CEVd is closely related to their homologous sequences. The right homologous region is the core region of abundant vd-siRNA production, and vd-siRNA from the pre-inoculated CBCVd may disrupt the replication and movement of CEVd through RNA silencing. Previous studies have shown that the TR region of viroids can bind to the plant nucleus, thus introducing proteins to promote viroid replication and movement [86,87]. Pre-inoculated CBCVd shares the homologous TR region with CEVd, resulting in reduced host symptoms and decreased CEVd titer compared to “Etrog” citrons inoculated with CEVd alone. In addition, the distribution patterns of vd-siRNAs in the TR region of the two viroids are similar. Vd-siRNA is one of the core effectors of RNA silencing in plant defense mechanisms. However, little is currently known about the role of vd-siRNA and related RNA-silencing genes in antagonizing citrus viroids. According to reports, the expression of RNA silencing genes *DCL2*, *RDR1*, *AGO2*, *AGO7*, and *silencing defective 3* (*SDE3*) are upregulated in citrus plants infected with CEVd alone [55]. The genes upregulated in CBCVd single infection and CEVd co-infection also include *DCL2* and *SDE3*. The *SDE3* gene encoding RNA helicase in *Arabidopsis* is associated with the production of secondary siRNAs for PTGS [88,89]. CBCVd can attenuate the symptoms and accumulation of CEVd through the host’s RNA silencing mechanism. These studies enrich our understanding of the interactions among citrus viroids and provide new ideas for creating citrus germplasm resistant to severe viroid diseases.

## 6. The Synergistic Effect among Citrus Viroids

In addition to cross protection, there is another interaction among citrus viroids in plants infected with different viroids, which can lead to more severe symptoms than pure additive effects. The presence of viroid mixtures in the host determines the antagonistic or synergistic relationship among citrus viroids. Citrus viroids often exhibit mixed infection during long-term asexual reproduction in the field [10]. Synergistic interactions have been observed in trees infected with certain combinations of viroids. Duran-Vila et al. [90] were the first to report that the complex infection of multiple viroids can worsen symptoms in the indicator plant. Serra et al. [9] confirmed through strict invasive cloning experiments that citrus viroids of the *Apscaviroid* genus can significantly enhance the symptoms in “Etrog” citron, which is a typical synergistic effect. The co-infection of citrus trees grown in the field with CBLVd and CVd-V, or with CDVd and CVd-V, has also been confirmed to produce synergistic effects among viroids, leading to more severe symptoms than simple superimposition [9,10]. Although there was no significant change in viroid titer, enhanced leaf symptoms and significantly stunted plants were observed [9]. Despite the lack of CEVd, the combination of viroid infections can still cause significant symptoms of exocortis disease on citrange rootstocks, further confirming that the synergistic effect among CBLVd, CDVd, and CBCVd is the cause of cracking symptoms [91]. In addition, the mixture of CBLVd and CDVd can also enhance symptom expression in citron plants. Surprisingly, there is also a synergistic effect when CBCVd and HSVd co-infect citrus [10]. When CBCVd and HSVd coexist, they can induce bark cracking on the trifoliate orange rootstocks, but the presence of additional viroids can limit this synergistic effect. On the contrary, the synergistic effect between CEVd and CBLVd is further enhanced by the presence of additional viroids [10].

Some researchers have shown that the synergistic effect of viruses is mainly due to different viruses encoding different silencing suppressors, which act on different steps of endogenous gene silencing pathways in plants, thereby exacerbating the impact on normal plant growth and development [92]. PTGS has a regulatory effect on plant development and defense responses, and the PTGS pathway that regulates defense and development has some common components. The co-infection of two different viruses can lead to an increase in symptom expression, mainly due to their silencing suppressors acting on different sites of the RNA silencing pathway [92,93]. Because viroids do not encode silencing suppressors, similar explanations cannot be used to explain the synergistic effects among viroids. There is also evidence that plant RNA viruses inhibit RNA silencing due to their involvement in the chelation of replication enzymes associated with siRNA and miRNA biogenesis. Viroids may also interfere with the host’s RNA silencing mechanism through similar mechanisms, thereby affecting symptom expression. The synergistic effect among distant viroids may be caused by more than one component that affects this mechanism. In addition, a study has shown that there is also a synergistic effect between viroid and plant virus [94]. The simultaneous infection of Peach latent mosaic viroid (PLMVd) and Prunus necrotic ringspot virus (PNRSV) can synergistically affect the host transcriptome of infected peach fruits, and frequent mixed infections under field conditions can lead to more complex transcriptional responses than single infections. It is reasonable to speculate that the synergistic effect among different viroids may also be due to the utilization of different components in the host’s body, affecting the complex transcription process of the host. When multiple types of viroids are co-infected, multiple components in the host’s body may be affected, leading to synergistic effects.

## 7. Prospect

As a natural host for at least eight species of viroids, citrus plants provide a natural platform for studying interactions among viroids. The antagonistic effect among citrus viroids intuitively leads to a decrease in the pathogenicity of the viroids, while the synergistic effect leads to an increase in the pathogenicity of the viroids. The antagonism and synergy among citrus viroids are similar to the internal strife and unity in the animal world. During internal battles, the attacking power is weakened, and the pathogenicity is weakened. When united, the attacking power is strengthened, and the pathogenicity becomes stronger. The interactions among citrus viroids are complex and interesting, making it worthwhile for researchers to invest more time and effort in future work.

As a biological stress response, viroid infection can induce the expression of RDR1, and plant RDR1 was first isolated and discovered through viroid induction [95]. The latest research has found that CEVd infection can induce the expression of DCL4 and RDR1 in tomatoes [96]. HSVd infection induced the production of ribosomal RNA (rRNA) precursors in cucumber, thereby inducing a large amount of 21 nt siRNA derived from rRNA, which was correlated with the methylation level in this region [46]. Infection with citrus viroids is likely to induce the expression of citrus RDR1, thereby inducing endogenous siRNA dependent on RDR1. Citrus samples are often co-infected with several citrus viroids. Compared with the small RNA sequencing results of healthy citron leaves, it was found that the proportion of endogenous 24 nt sRNAs decreased significantly in viroid-infected samples, while the proportion of endogenous 21 nt sRNAs increased significantly. The co-infection of viroids may induce the accumulation of endogenous 21 nt miRNAs and the decrease in 24 nt siRNAs in plants. Citrus viroids may affect the endogenous gene-silencing system of plants. Through differential expression and functional analysis of target genes and gene silencing pathway genes (such as *DCL*, *RDR*, *AGO*, etc.), it is expected to clarify the molecular mechanisms of synergistic effects of citrus viroids in the future.

Small RNAs derived from viroids play a crucial role in the pathogenicity of viroids [97,98]. The identification of sRNA of citrus viroids is of great value for understanding the interaction mechanism among citrus viroids. The work of Qiu et al. [85] has demonstrated that CBCVd attenuates the symptoms and accumulation of CEVd through the host’s RNA silencing mechanism. The antagonistic mechanism among citrus viroids is complex, and more experimental data are needed to verify the core position of vd-siRNA in the antagonistic mechanism of citrus viroids. Based on current experience, CBCVd and CEVd can produce a large number of identical vd-siRNAs in the homologous region when infecting citrus. When CBCVd and CEVd co-infect citrus, the homologous region is the hotspot region for vd-siRNA production. The mechanism by which homologous regions can produce a large number of identical vd-siRNAs also needs further clarification. In addition, Wang et al. [23] found that CBCVd-lss in Pakistan showed a decrease in homology with the right-handed end region of CEVd compared to the CBCVd reference. Further experiments are needed to clarify whether there is still antagonism between CBCVd-lss and CEVd when they co-infect citrus, and whether they can still produce large amounts of vd-siRNA in the homologous region. These works are crucial for further clarifying the antagonistic mechanisms among citrus viroids. It is worth noting that there is an antagonistic effect between CBCVd and CEVd in citrus, while there is a synergistic effect between CBCVd and HSVd [10]. CBCVd is homologous with HSVd in the left terminal region, and highly homologous with CEVd in the right terminal region. These phenomena indicate that the interaction among citrus viroids is very complex. The performance of the final interaction results may be influenced by multiple factors such as the viroid sequence, viroid structure, and host species. The elucidation of the interaction among citrus viroids is still a long and arduous task.

## 8. Conclusions

There are complex and interesting interactions among citrus viroids, making citrus the best carrier for studying viroid interactions. Specifically, there are antagonistic and synergistic phenomena with the antagonistic effect intuitively leading to a decrease in the pathogenicity of citrus viroids, while synergism increases the pathogenicity. We have summarized the main progress made in citrus viroids in recent years, focusing on the antagonistic and synergistic phenomena observed, and pointing out the core roles of host RNA silencing and vd-siRNA in citrus viroid interactions. Our review provides suggestions for future research directions on citrus viroid interactions, hoping to encourage more people to participate in this interesting and meaningful study.

## Data Availability

Not applicable.
